# Detection and Quantitation of Gluten in Fermented-Hydrolyzed Foods by Antibody-Based Methods: Challenges, Progress, and a Potential Path Forward

**DOI:** 10.3389/fnut.2019.00097

**Published:** 2019-06-28

**Authors:** Rakhi Panda, Eric A. E. Garber

**Affiliations:** Division of Bioanalytical Chemistry, Office of Regulatory Science, Center for Food Safety and Applied Nutrition, United States Food and Drug Administration, College Park, MD, United States

**Keywords:** gluten, fermentation, quantitation, competitive ELISA, hydrolysis, peptides

## Abstract

Celiac disease (CD) affects ~1 in 141 individuals in the United States, requiring adherence to a strict gluten-free diet. The Codex Standard and the European Commission states that gluten level of gluten-free foods must not exceed 20 ppm. The FDA requires food bearing the labeling claim “gluten-free” to contain <20 ppm gluten. Accurate quantitation of gluten in fermented-hydrolyzed foods by antibody-based methods is a challenge due to the lack of appropriate reference materials and variable proteolysis. The recent uses of proteases (e.g., proline endopeptidases or PEP) to hydrolyze immunopathogenic sequences of gluten proteins further complicates the quantitation of immunopathogenic gluten. The commercially available antibody-based methods routinely used to detect and quantitate gluten are not able to distinguish between different hydrolytic patterns arising from differences in fermentation processes. This is a severe limitation that makes accurate quantitation and, ultimately, a detailed evaluation of any potential health risk associated with consuming the food difficult. Utilizing gluten-specific antibodies, a recently developed multiplex-competitive ELISA along with western blot analysis provides a potential path forward in this direction. These complimentary antibody-based technologies provide insight into the extent of proteolysis resulting from various fermentation processes and have the potential to aid in the selection of appropriate hydrolytic calibration standards, leading to accurate gluten quantitation in fermented-hydrolyzed foods.

## Introduction

Celiac disease (CD) is an immune mediated enteropathy triggered by the interaction of the prolamin and glutelin fractions of proteins from wheat, barley, and rye with the intestinal mucosa of sensitive individuals (1). Upon ingestion, proteases in the gastrointestinal tract degrade gluten proteins into peptides, which undergoes deamidation by transglutaminase. Subsequently, these peptides interact with human leukocyte antigen (HLA)-DQ2 or -DQ8 molecules evoking a T cell response, resulting in inflammation in the small intestine (2, 3). Gluten can be fractionated into alcohol soluble prolamins and the alcohol insoluble glutelins. The wheat prolamins, gliadins, are monomeric proteins with molecular weight ranging from 30 to 50 kDa and can be classified into α/β, γ, and ω-type. The wheat glutelins, glutenins, can be divided into high molecular weight (HMW) glutenins with molecular weights of 66–88 kDa, and low molecular weight (LMW) glutenins with molecular weights falling in the range of the gliadin proteins, ~32–45 kDa (4, 5). A typical feature of gluten T cell stimulating peptides is their high proline content. Proline constitutes 12–17% of gluten. The abundance of proline residues in gluten makes them highly resistant to complete proteolytic degradation in the human gastrointestinal track (6, 7).

Approximately 1 in 141 people in the US are affected by CD and adherence to a strict gluten-free diet is the only option to prevent inflammatory symptoms in sensitive individuals (8, 9). In 2013, the FDA issued a regulation defining and allowing the use of the term gluten-free for food that “does not contain an ingredient that is a gluten-containing grain (e.g., spelt wheat); an ingredient that is derived from a gluten-containing grain and that has not been processed to remove gluten (e.g., wheat flour); or an ingredient that is derived from a gluten-containing grain and that has been processed to remove gluten (e.g., wheat starch), if the use of that ingredient results in the presence of 20 parts per million (ppm) or more gluten in the food [i.e., 20 milligrams (mg) or more gluten per kilogram (kg) of food]; or inherently does not contain gluten; and that any unavoidable presence of gluten in the food is below 20 ppm gluten (i.e., below 20 mg gluten per kg of food).” It was further “recognized that some food matrices, such as fermented or hydrolyzed foods, may lack currently available scientifically valid methods that can be used to accurately determine if these foods contain ≥20 ppm gluten” (10). Recognizing the unique problems associated with the accurate detection and quantitation of gluten in fermented foods, a regulation regarding the use of gluten-free label for fermented, hydrolyzed, and distilled foods was proposedin 2015 (11).

Several qualitative and quantitative analytical methods are used for the detection and quantitation of gluten in foods. The strengths and limitations of each method have been summarized in Table 1 (12–15). Enzyme-linked immunosorbent assays (ELISAs) are currently the most popular method used to detect and quantitate gluten in foods. Most commercial ELISAs for gluten quantitation employ monoclonal antibodies such as Skerritt, R5 and G12. A polyclonal antibody against gluten proteins is also available from the Morinaga Institutes of Biological Sciences, Inc., (MIoBS). The Skerritt antibody was raised against wheat gliadin and has been shown to recognize the HMW glutenins (16–18). The R5 antibody was raised against rye secalin and strongly binds to the QQPFP, QQQFP, LQPFP, and QLPFP epitopes in α-/β-, ω-, and γ-gliadins (19, 20). The G12 antibody was produced against a synthetic 33-mer (LQLQPFPQPQLPYPQPQLPYPQPQLPYPQPQPF) of α2-gliadin, believed to invoke immunopathogenicity and the antibody recognizes the QPQLPY epitope of the peptide (21, 22). Recently, a novel monoclonal antibody that recognizes deamidated gliadin was generated by Pi Bioscientific Inc. (23, 24). Detection and quantitation of intact gluten has been routinely performed using sandwich ELISAs (16–19, 21, 25–29). There are questions related to accuracy of the results with respect to antibody specificity, extraction procedure, lack of suitable reference materials as well as of scientific data to support the underlying assumptions for calculating the gluten content, that has been extensively reviewed in several previous publications (9, 30–32).

**Table 1:** Common analytical techniques used for detection of gluten in foods.

**Common gluten detection techniques**	**Strengths**	**Limitations**
Sandwich ELISA	- Commercially available - Specific - Sensitive - Robust - Quantitative analysis of intact gluten is possible	- Not suitable for quantitation of fermented-hydrolyzed gluten - Lack of certified reference materials limit the accuracy of the results
Competitive ELISA	- Commercially available - Appropriate for fermented-hydrolyzed gluten	- Usually less sensitive and robust compared to sandwich ELISA - Appropriate calibrant is needed for accurate analysis results
Immunosensors/ Dipsticks/Lateral flow devices (LFDs)	- User friendly - Rapid analysis - Useful for on-site analysis - Commercially available	- Usually qualitative or semi-quantitative
Western blots	- Separates and detects gluten proteins according to their size - Can be used as a confirmatory technique for ELISA	- Less sensitive compared to ELISAs - Not commercially available - Requires expertise - Usually qualitative/semi-quantitative
Mass spectrometry	- Highly sensitive - Can directly detect proteins/peptides that are not detected by immunological techniques - Quantitative analysis is possible	- Expensive equipments - Requires expertise - Similar to the ELISAs need certified reference materials for accurate quantitation - Depends on publicly available databases of wheat and barley proteins, which in most cases are incomplete or are poorly curated
DNA-based methods	- Stable analyte - DNA is more efficiently extracted compared to proteins - Can be used as a highly sensitive screening method for the presence of gluten containing cereals - Quantitative analysis is possible using quantitative real-time PCR (Q-PCR)	- Unsuitable for highly processed or fermented-hydrolyzed foods
Aptamer-based assays	- New generation methods - Highly sensitive	- Extensive validation studies are lacking in different food matrices

The reliable detection and accurate quantitation of gluten in fermented-hydrolyzed foods is another challenge that warrant further discussion. This review will discuss the challenges involved in the detection and quantitation of fermented-hydrolyzed gluten by antibody-based methods and a potential path forward in overcoming the challenges. Although significant progresses have been made by using mass spectrometry-based methods in this direction, this review will only discuss mass spectrometry-based methods briefly and will particularly focus on antibody-based methods.

## Different Fermentation Processes and Gluten Protein/Peptide Profile Differences

Cereal-based fermented-hydrolyzed foods can be classified into different categories depending on the grain source, type of fermenting organism, and differences in the fermentation process. Wheat, rye and barley are commonly used in fermented-hydrolyzed foods such as beers, soy sauces, vinegars, and sourdough breads.

### Beers

Beer is the most widely consumed alcoholic beverage made from celiac-toxic cereals, mainly barley, and wheat. During mashing, malting, and fermentation, the gluten is proteolyzed by enzymes, resulting in the formation of peptides. Gluten-derived peptides tend to remain in the final beer product and often contain immunopathogenic sequences (33). Studies have detected peptide fragments from the putative immunotoxic 33-mer of α2-gliadin in several wheat and barley beers produced by different manufacturing processes, indicating the resistance of this peptide to proteolytic cleavage during the production of beers (17, 34, 35). The susceptibility of different gluten proteins to proteolysis during fermentation varies, thereby generating a very diverse range of peptides. A study by Colgrave et al. (36), indicated that B-hordein and D-hordein are more susceptible to hydrolysis compared to γ-3 hordeins. In recent years, several mass spectrometry studies have detected and characterized gluten proteins/peptides in both wheat and barley beers (17, 34–43).

### Soy Sauces

Soy sauces are popular fermented foods that are commonly used to impart flavor. Soy sauce is produced in a two-stage fermentation process of soybean and wheat. First, koji (a mold-covered mixture of soybeans and wheat) is generated, which is mixed with salt water to form moromi. The moromi is allowed to age for several months, during which fermentation is catalyzed by lactic acid bacteria and yeast (44). Several studies have indicated the absence of any gluten-derived peptides in soy-based sauces using ELISAs or serum IgE binding studies (45–47), which is consistent with the extensive proteolysis that occurs during soy sauce fermentation. Although soy sauces produced by classical fermentation may lack the presence of gluten derived proteins/peptides, any changes to the fermentation process, or ingredients used may alter the extent and type of proteolysis and, possibly, the immunopathogenicity. A study by Hefle et al. (48) indicated that some soy sauces contained 10–30% residual activity by means of RAST inhibition assays using sera from soy-allergic subjects (48). A recent western blot study indicated the presence of gluten-derived proteins/peptides in several soy-based sauces. Intact gluten was detected in a teriyaki sauce and gluten-derived peptides were detected in one soy sauce and two Worcestershire sauces. The exact quantity of gluten in these products could not be ascertained from the immunoblot data; however, the detection of gluten-derived proteinaceous materials in these products indicate the potential for immunopathgenicity (49).

### Vinegars

Malt vinegars are produced by fermentation of cereals containing gluten, mostly barley and wheat. Vinegars made from distilled ethanol, are generally produced from non-gluten-containing raw material such as corn, beet, or sugar cane, but in some cases also gluten-containing cereals. The raw materials are typically processed in a manner that avoids the presence of any non-volatile compounds (e.g., gluten) from the finished product. However, exceptions to this occur when the distillation process is poorly performed. Thus, it is not uncommon to observe gluten peptides in some vinegars (44). Gluten peptides have been detected in vinegars both in western blot as well as mass spectrometry studies (43, 49, 50). Further, immunopathogenic epitopes in the HMW glutenin peptides derived from a malt vinegar have been reported. However, it is unclear whether the amount of glutenin present is sufficient to pose a health risk for celiac patients (50).

### Sourdough Breads

Sourdough is a mixture of flour (usually wheat and/or rye), water, and other ingredients that are fermented by naturally occurring lactic acid bacteria and yeasts. The potential of sourdough lactic acid bacteria as a source of proteolytic enzymes has also been investigated recently. Although primary proteolysis during sourdough fermentation is exerted by wheat or rye endogenous enzymes that are activated by the low pH, studies have shown that certain strains of lactic acid bacteria used in sourdough fermentation can produce peptidases that can proteolytically cleave the gliadin fraction of wheat gluten under certain conditions (51–54). However, as was observed in the production of beers, the glutenin fraction of gluten has been shown to be more resistant to microbial proteolysis, so sourdough breads can still pose a potential health risk for those with celiac disease (53, 54). Further, a study has shown that lactic acid fermentation of wheat flour does not degrade gluten sufficiently enough to decrease available transglutaminase 2 binding sites on α2-gliadin and, therefore, doesn't prevent the interaction of enzyme transglutaminase 2 with gluten, indicating another source of potential immunopathogenicity (55).

### Protein/Peptide Profile Differences

Quantitation of gluten in fermented-hydrolyzed foods poses a challenge due to lack of methods that can recognize the highly variable proteolytic peptide patterns that vary between fermentation processes, as well as due to the lack of suitable hydrolytic calibrants. This is further complicated by the lack of clinical information correlating peptide content with biological activity. Further, it is unknown how to interpret the immunopathogenicity based on the amount or profile of gluten protein/peptides being detected in several different fermented-hydrolyzed foods. The regulatory threshold of 20 ppm intact gluten was based on studies examining the immunopathogenicity of intact gluten. Whether the biological activity is the same for gluten peptides that are produced during fermentation is unknown (29, 56–59).

**Figure 1:**
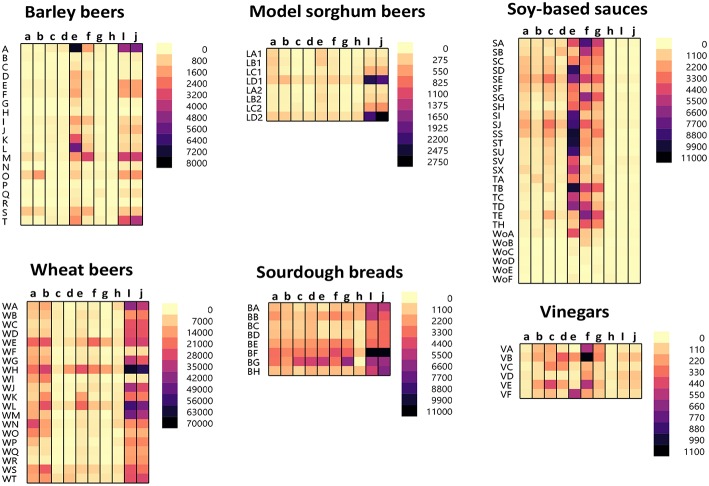
Apparent gluten concentration (μg/mL) profiles of different categories of fermented-hydrolyzed foods (20 barley beers, 20 wheat beers, 8 model sorghum beers containing 200 μg/mL gluten brewed in the presence and absence of PEP, 8 sourdough breads, 27 soy-based sauces, and 6 vinegars) as analyzed by the multiplex-competitive ELISA utilizing gluten specific antibodies from the AgraQuant Gluten G12 (a), GlutenTox ELISA Competitive G12 (b), RIDASCREEN Gliadin (c), RIDASCREEN Gliadin Competitive (d), Veratox for Gliadin, cat # 8480 (e), Neogen Veratox for Gliadin R5 (f), Microbiologique Gluten (g), Morinaga Institute of Biological Sciences, Inc. (MIoBS) Gliadin (h), and AllerTek Gluten (25 (i) and 10 μg/mL (j), respectively coating antigen concentration) ELISA kits (56). For soy-based sauces, SA-SX represents soy sauces, TA-TH represents teriyaki sauces, and WoA-WoF represents Worcestershire sauces. For the model sorghum beers, LA-LC represent 200 ppm gluten containing beers brewed in the presence of different concentrations of PEP (25, 4, and 0.5 mL/31 gallon of wort, respectively) and LD represents 200 ppm gluten containing beer brewed in the absence of PEP. LA1-LD1 and LA2-LD2 represents two different replicate brews.

The protein/peptide profile generated during the fermentation of different foods is dependent on numerous parameters. These include the ingredients used, time, temperature, and fermenting organisms. A slight change in these parameters can lead to wide variations in the protein/peptide profile. As such, it is impossible to generalize the profile for the different fermentation processes. The protein/peptide profiles of different fermented foods were examined using a recently developed multiplex-competitive ELISA. The ELISA utilized HRP (Horseradish peroxidase)-conjugated gluten specific antibodies (G12, R5, 2D4, MIoBS, and Skerritt) from nine gluten ELISA test kits. The antibodies were utilized in a competitive ELISA format by multiplexing the nine gluten specific antibodies into a single assay plate as described previously (56). Figure 1 shows the apparent gluten concentration values obtained for six different fermented-hydrolyzed food categories using the multiplex-competitive ELISA. Included in the analysis were barley beers, wheat beers, a model sorghum beer brewed with 200 ppm gluten (added prior to fermentation) and brewed in the presence and absence of a PEP (Brewers Clarex), sourdough breads, soy-based sauces (soy sauces, teriyaki sauces, and Worcestershire sauces), and vinegars. Since, the antibodies used in the multiplex-competitive ELISA displays different specificities (gliadin, glutenin, and deamidated gliadin), the profiles reflect the antigenic differences arising due to the different manufacturing processes. As illustrated in Figure 1, the protein/peptide profile as recognized by the different gluten specific antibodies varied among the different categories of fermented-hydrolyzed foods. For example, comparing the wheat and the barley beers, the apparent gluten concentration values of the wheat beers using all the nine antibodies were higher than the barley beers. Higher gluten content has been observed in wheat beers compared to barley beers in several previous studies (35, 38, 41, 60, 61). Further, by western blot, higher level of immunoreactive peptides have been identified in wheat beers compared to barley beers (35). Another interesting difference that was observed between the profiles of the wheat beers and the barley beers, using the multiplex-competitive ELISA, was the higher apparent gluten concentration values using the two G12 antibodies (a and b) in wheat beers compared to barley beers. This observation is consistent with a previous study, which showed high level of 33-mer equivalent peptides (specifically recognized by the G12 antibodies) in wheat beers compared to the barley beers (34). Further, the wheat beers, the model sorghum beers brewed with 200 ppm gluten, and the sourdough breads resulted in a comparatively high apparent gluten concentration by the Skerritt antibody (i and j), indicating the possible abundance of glutenin proteinaceous materials. However, this was not the case with the soy-based sauces and vinegars, which instead resulted in comparatively high apparent gluten concentration values with both the Neogen antibodies (e and f) and the Microbiologique gluten antibody (g), indicating a relatively higher abundance of gliadin, and deamidated gliadin. These results indicate that protein/peptide profile differences exist among various fermentation processes. Further, the recognition of the protein/peptide profile differences, as achieved by the multiplex-competitive ELISA, is not possible if a single gluten-specific antibody is used in an assay for the detection of gluten, which is usually the case with the commercially available ELISA kits. This limits the utility of the commercial ELISAs in accurately quantitating gluten in several different types of fermented-hydrolyzed foods. The recognition of the differences in the proteolytic patterns among the different fermentation processes by a gluten detection assay is essential for the selection of appropriate calibration standards of comparable digestion and similar peptide composition, leading to accurate quantitation of gluten in different categories of fermented-hydrolyzed foods.

## Proline Endopeptidases (PEP) to Reduce Immunopathogenic Gluten Content

Several proteases [PEP derived from *Aspergillus niger* (AN-PEP)*, Sphingomonas capsulate*, EP-B2 (cysteine endoprotease from germinating barley), ALV003 (mixture of cysteine endoprotease and PEP), and Pseudolysin (lasB)] have been recently used to enzymatically hydrolyze gluten proteins in an attempt to prevent proliferative responses in gluten specific T cells (58, 62–70). The *Aspergillus niger* derived PEP (AN-PEP) and the ALV003 have been evaluated in clinical trials for their effectiveness in mitigating gluten-induced immune responses in celiac patients (71, 72). PEP is a serine protease which proteolyzes the peptide bonds at the carboxyl end of prolines. The use of AN-PEP in hydrolyzing gluten present in wheat starch, wheat bran, and a non-alcoholic cereal-based beverage has been reported (73, 74). In the manufacture of beer, AN-PEP, commercially available as Brewers Clarex, has been frequently used to prevent chill-haze formation that involves hydrophobic interaction of polyphenols with proline-rich proteins in beer. This enzyme has an optimum pH around 4.5, making it suitable for use during fermentation to brew beer (75). There are several conflicting reports on the ability of AN-PEP to sufficiently proteolyze gluten and eliminate any immunopathogenicity. A study by Guardum and Bamforth indicated that addition of PEP during brewing process reduced the prolamin contents of beers (76). A mass spectrometric study also reported that AN-PEP was effective in eliminating all known immunopathogenic gluten epitopes during beer production (77). However, not all potentially immunopathogenic sequences were monitored in the study. A third study indicated that PEP could destroy gluten T-cell epitopes (64). In contrast, several recent studies utilizing mass spectrometry, ELISA, and western blot analysis indicated that PEP didn't completely degrade all gluten proteins and gluten proteins/peptides remain in the final beer produced by addition of PEP. Specifically, the HMW glutenin were resistant to the action of PEP during beer production (17, 39, 78). In addition, beer treated with PEP has been shown to cause a humoral response toward IgA or IgG antibodies, derived from the sera of 3 celiac disease-active patients, but there was no response from normal control subjects (*n* = 31, control group: *n* = 29), indicating that beers treated with PEP are still immunogenic (79). In another mass spectrometry study, gluten peptides that contained sequences associated with celiac disease were detected in a model wheat containing sorghum beer brewed in the presence of PEP. Included among the peptides detected were the LQLQPFPQPQLPY peptide, which is the beginning of the immunopathogenic 33-mer, and hydrolyzed HMW glutenin peptides containing immunogenic sequences (39).

**Figure 2:**
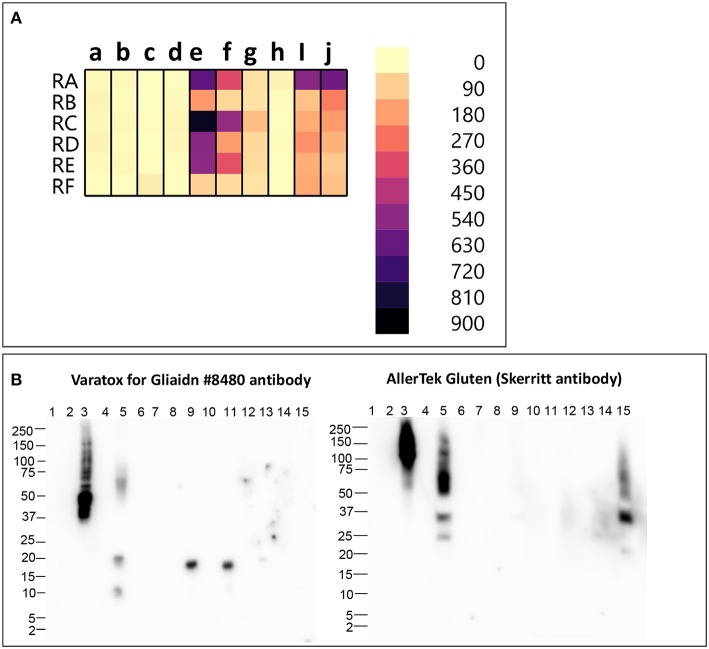
**(A)** Apparent gluten concentration (μg/mL) profiles of gluten -reduced barley beers (RA, RB, RC, RD, RE, RF), obtained by the multiplex-competitive ELISA utilizing gluten specific antibodies from the AgraQuant Gluten G12 (a), GlutenTox ELISA Competitive G12 (b), RIDASCREEN Gliadin (c), RIDASCREEN Gliadin Competitive (d), Veratox for Gliadin, cat # 8480 (e), Neogen Veratox for Gliadin R5 (f), Microbiologique Gluten (g), Morinaga Institute of Biological Sciences, Inc. (MIoBS) Gliadin (h), and AllerTek Gluten (25 (i) and 10 μg/mL (j) coating antigen concentration) ELISA kits (56). **(B)** Western blot binding signal of gluten-reduced barley beers (RA, RB, RC, RD, RE, RF), using the detector antibodies of Veratox for Gliadin, cat # 8,480 and AllerTek Gluten ELISA kits. Lane information for western blot: Lane 1- Molecular weight marker, Lane 2- Empty, Lane 3- 2.5 μg/mL intact gluten standard, Lane 4- Empty, Lane 5- beer RA, Lane 6- Empty, Lane 7- beer RB, Lane 8- Empty, Lane 9- beer RC, Lane 10- Empty, Lane 11- beer RD, Lane 12- Empty, Lane 13- beer RE, Lane 14- Empty, Lane 14- beer RF (49).

We analyzed six different commercial gluten-reduced beers (brewed in the presence of PEP to reduce their gluten content) using the multiplex-competitive ELISA and western blots (49, 56). The apparent gluten concentration measured by the multiplex competitive ELISA was high for all the gluten-reduced beers with at least one gluten specific antibody (Figure 2A). Specifically, the Skerritt antibody and the two Neogen Varatox antibodies resulted in high apparent gluten concentrations with multiple gluten-reduced beers (Figure 2A). Although R5 antibodies from two other ELISA kits, RIDASCREEN gliadin (c) and RIDASCREEN gliadin competitive (d), were used in the multiplex-competitive ELISA, the apparent gluten concentration values with those antibodies were much lower compared to the Neogen Veratox R5 antibody (f). Differences in the sensitivity displayed by the same antibody derived from different test kits can be easily explained by differences in HRP conjugation resulting in higher catalytic activity. This leads to the question on a more complex issue of why differences in the performance of the same antibody in two different ELISA test kits arise. It could be due to differences in the handling of the antibody, such as in the coating of the microtiter plates and the chemistry associated with HRP conjugation altering the binding properties (affinity) toward the target analyte. More complex differences, that may alter the performance relative to defined calibration standards may arise from changes to the binding conditions, including the coating of the microtiter plates to block non-specific interactions. Lastly, all quantitative analyses are dependent on the calibration standards employed. In as much as there are no universally recognized gluten standards that are employed by all test kit manufacturers, it is possible that two kits employing identically prepared antibody reagents may differently calculate gluten content (9, 31, 80, 81).

In the western blot, 3 gluten-reduced beers, RA (Lane 5), RC (Lane 9), and RD (Lane 11), resulted in bands with the Veratox for Gliadin, cat # 8,480 detector antibody (Figure 2B). Bands at 17 kDa in beers RC (Lane 9) and RD (Lane 11) represent gluten-derived peptides, whereas binding observed to multiple protein bands at 10, 20, and 50–75 kDa in beer RA (Lane 2) indicate both intact gluten and gluten-derived peptides (Figure 2B). Beer RA is produced by a different manufacturer than beers RC and RD. Therefore, the differences in band pattern observed can be attributed to the differences in the manufacturing processes employed by the two companies. Nevertheless, gluten proteins/peptides remain in the final products, confirming the findings of previous mass spectrometry studies (36, 39). With the Skerritt antibody, beers RA (Lane 5) and RF (Lane 15) yielded multiple bands (20–150 kDa) both at higher and lower MW range (Figure 2B). Binding to Skerritt antibody indicates the presence of HMW glutenin (D Hordein) epitopes in gluten-reduced beers and again confirms the results of previous studies (17, 39, 78). The presence of HMW glutenin in gluten-reduced beers may not get accurately detected by gluten detection assays targeting only gliadin proteins. Further, studies have indicated that glutenin proteins can develop toxic response in celiac patients. Therefore, consumption of gluten-reduced beers may pose a potential concern for individuals with CD (17, 39, 82–84).

## Commercially Available ELISA Methods Are Not Accurate for Fermented/Hydrolyzed Gluten

Table 2 lists the various commercial ELISAs that are routinely used for the detection and quantitation of gluten in foods. ELISAs in both sandwich and competitive format are available. Sandwich ELISAs require two epitopes and therefore cannot detect short peptides lacking two antibody binding sites. However, celiac disease requires only a single immunopathogenic element, thereby making it possible for sandwich ELISAs to miss toxic gluten-derived peptides in fermented-hydrolyzed foods (17, 29). In contrast, competitive ELISAs recognize a single epitope and may be more effective in detecting immunopathogenic peptides derived from gluten in fermented-hydrolyzed foods. Competitive ELISAs based on R5 (RIDASCREEN® Gliadin Competitive) and G12 (GlutenTox® Competitive) monoclonal antibodies are marketed for detection and quantitation of gluten in fermented-hydrolyzed foods.

**Table 2:** Manufacturer's specified properties of commercially available gluten ELISA test kits.

**ELISA kits**	**Manufacturer**	**Target**	**Capture**	**Detector**	**LOD^a^**	**LOQ^a^**	**Upper limit^a^**
AgraQuant ELISA Gluten G12	Romer Labs	QPQLPY	G12 monoclonal	G12 monoclonal	2	4	200
GlutenTox ELISA Competitive	Biomedal Diagnostics	QPQLPY	Gliadin	G12 monoclonal	–	3	48
RIDASCREEN Gliadin Sandwich	R-Biopharm, AG	QQPFP, QQQFP, LQPFP, QLPFP	R5 monoclonal	R5 monoclonal	3	5	80
RIDASCREEN Gliadin Competitive	R-Biopharm, AG	QQPFP, QQQFP, LQPFP, QLPFP	Gliadin	R5 monoclonal	2.6	10	270
Veratox for Gliadin, 8480	Neogen Corp.	Gluten	USDA monoclonal^b^	USDA monoclonal^b^	–	5	50
Veratox for Gliadin R5	Neogen Corp.	QQPFP, QQQFP, LQPFP, QLPFP	R5 monoclonal	R5 monoclonal	–	5	80
AllergenControl ^TM^ Gluten Sandwich	Microbiologique Inc.	Gliadin	2D4^c^	2D4^c^	-	2.5	80
Wheat Protein ELISA (MIoBS)	Morinaga Institute of Biological Sciences, Inc.	Gliadin	Polyclonal	Polyclonal	0.24	0.25	16
AllerTek Gluten	ELISA Technologies, Inc.	HMW^d^ glutenin	Skerritt monoclonal	Skerritt monoclonal	–	5	80
GlutenTox ELISA Sandwich	Biomedal Diagnostics	QPQLPY	A1 monoclonal	A1 monoclonal	–	0.6	10

a *Expressed as mg/kg (ppm) gluten*.

b *Gluten specific monoclonal antibody developed and licensed by the U.S. Department of Agriculture (USDA)*.

c *Deamidated gliadin specific antibody*.

d *HMW, High molecular weight*.

The RIDASCREEN® R5 competitive ELISA utilizes a mixture of pepsin-trypsin digested prolamin fractions from wheat, rye, and barley as the calibrator for quantitation purposes. Though awarded First Action by the Association of Official Analytic Chemists Official Methods of Analysis (AOAC OMA) (59, 85), the validation of this method was based on the detection of the reference material spiked into various foods and the AOAC OMA specifically states that depending on the fermentation conditions and resulting proteolysis, the validation performed may not be scientifically valid. It is critical that the calibration standards reflect the peptides produced by the proteolysis and the appropriate amount of residual intact gluten. Further, to be representative of real-world samples, the analyte must be incurred prior to processing (86, 87).

**Figure 3:**
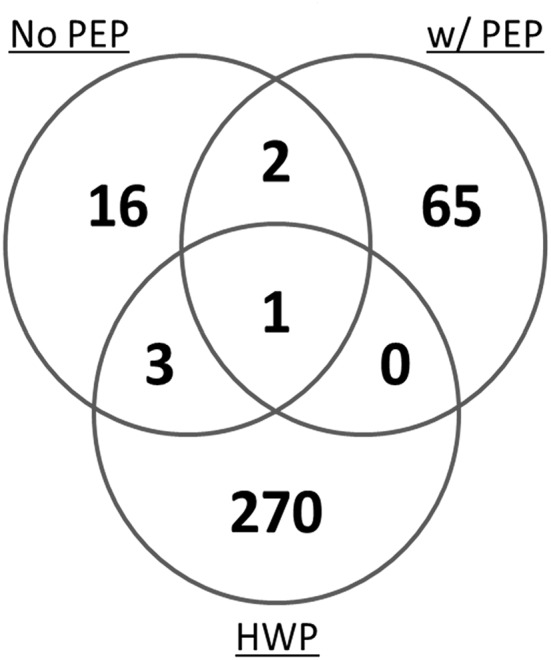
Venn diagram of the total number of unique gluten peptides identified by mass spectrometry in a model sorghum beer containing 200 μg/mL gluten, brewed in the presence and absence of PEP, comparing it to that of the HWP. The samples were analyzed in triplicate and only peptides that were identified in at least two of the three injections were included (17).

In a mass spectrometry study, we evaluated the potential of the hydrolyzed wheat prolamin (HWP, used as a calibrant in the R5 competitive ELISA) as a calibrant for the detection of gluten in a model sorghum beer containing 200 mg/L added gluten, brewed with or without the addition of PEP (17). By mass spectrometry, 274 unique gluten peptides were detected in HWP. However, only 4 peptides were represented in the peptide profile of a 200 mg/L gluten containing beer brewed without PEP and 1 was represented in that of the PEP containing beer (Figure 3). These disparities in the peptide profiles between HWP and the beers reflects the unsuitability of HWP as a calibrant for accurate quantitation of gluten in these beers. Although specific types of beers were brewed in the study, variability in fermentation conditions (time, temperature, pH) would likely result in a peptide profile not compatible with using HWP as a calibrant for accurate gluten quantitation.

Another limitation of the R5 competitive ELISAs is the use of gliadin as the calibrant without the inclusion of the glutenin fraction of gluten. Though the gliadin fraction of gluten is mainly responsible for exacerbating celiac disease, glutenin proteins have also been shown to stimulate celiac small intestinal T cells and can induce a toxic response in patients (83, 84). In another study, Tye-Din et al. (82) identified gluten T-cell stimulatory peptides that resembled the HLA-DQ8-restricted epitope present in HMW glutenin (82). Studies have shown that peptides derived from HMW glutenins (known as D hordein in barley) are present in beers (17, 39). The presence of HMW glutenin-derived peptides have also been reported in sourdough breads and vinegars (50, 53, 54). Therefore, calibration standards based on gliadin proteins are likely not be suitable for accurate quantitation of gluten in fermented-hydrolyzed products.

A G12 antibody based competitive ELISA is also available for the detection of gluten. Although, the G12 antibody was raised against a prominent immunogenic gluten peptide, it may not recognize all known potential immunogenic-sequence-containing gluten peptides. This is complicated by the fact that not all immunogenic sequences associated with CD is known due to incomplete understanding of the pathogenesis of CD (21, 22, 88, 89). Studies have indicated that in fermented beverages such as beer, the reactivity of the G12 antibody to peptides correlates with potential celiac immunotoxicity. T cell epitopes in beer have been recognized with the highest affinity by the G12 antibody (34, 90). Further, the G12 antibody has been shown to be more efficient at immunocapturing the T-cell active peptides from a barley beer and a hydrolyzed gliadin from wheat compared to the R5 antibody (91). This indicates that the G12-based ELISA may be more suitable for the analysis of fermented-hydrolyzed gluten compared to the R5-based ELISA. However, no information is available on the calibrant used in the G12 competitive ELISA and validation studies have not been performed to establish the reliability of the = ELISA nor its ability to accurately quantitate gluten in fermented-hydrolyzed foods.

## Recent Progress and a Potential Path Forward

Competitive ELISAs based on the G12 and R5 antibodies cannot distinguish between the protein/peptide profile pattern of different fermented foods, and while they target gliadins, they do not accurately detect glutenins, which also contain immunopathogenic sequences (17, 39, 83, 84). Mass spectrometry has the ability to differentiate the peptide profile differences among different fermentation processes (35, 36), thereby providing for a potential alternative to immunodiagnostic methods in developing suitable calibration standards for accurate quantitation of gluten in fermented-hydrolyzed foods. Semi-quantitation by mass spectrometry is possible by comparing mass areas measured from food samples against the appropriate calibration curve obtained by measuring mass areas of standard prolamin solutions (40). Recently, targeted approaches such as multiple reaction monitoring (MRM) mass spectrometry have been used for relative quantitation of gluten-derived tryptic peptides in fermented beverages such as beers (36, 92). In this method, multiple peptides are monitored per protein after trypsin digestion to compare the abundance of the original protein in different samples. MRM mass spectrometry combined with the use of synthetic peptide standards has been utilized for quantitation of six potentially immunopathogenic wheat gluten peptides in a range of native flours, processed products, sauces and beverages, including a light beer, and a vinegar (43). However, there are several limitations associated with using mass spectrometry as a quantitative method for routine analysis of gluten in fermented-hydrolyzed foods. A major limitation is dependence on identification of all the immunopathogenic sequences associated with celiac disease, however, such is not the case (88, 89). Further, similar to ELISA, accurate quantitation by mass spectrometry also requires suitable calibration standards, whereby the peptide content can be related to the regulatory threshold of 20 ppm intact gluten. Also, publicly available databases of plant protein sequences are incomplete, in particular for wheat and barley gluten proteins, further limiting the utility of mass spectrometry (36, 39).

Recognition of protein/peptide profile differences among different fermentation processes is the first step toward selection of appropriate calibration standards and, eventually, to the development of a method that can accurately quantitate gluten in fermented-hydrolyzed foods. Accurate quantitation requires that the calibration standard be identical to the protein/peptide profile of the fermented-hydrolyzed foods. This means encompassing all the gluten components (gliadin and glutenins, or any modifications resulting from fermentation, such as deamidated gliadin) present. A single calibration standard will not be suitable for all fermented-hydrolyzed foods. As such, any analytical method that is used to analyze multiple fermentation products must be able to distinguish between the different protein/peptide profiles so the appropriate calibrant can be selected to ensure accurate quantitation.

The novel multiplex-competitive ELISA included gluten specific antibodies from nine different commercial ELISA test kits. Utilizing antibodies that target different gluten epitopes, it was possible to distinguish between the protein/peptide characteristics of several different fermentation processes [Figure 1, (56)]. This assay simultaneously detects gliadin, deamidated gliadin and glutenin derived proteins, and peptides. Wheat gluten was used as a calibrant in the assay. Variability in the quantities and proportions of gluten proteins among wheat, rye, and barley cultivars exists and this makes the establishment of a universal standard or reference material problematic (93–96). Although reference materials comprised of both wheat gliadin and barley hordein have been proposed for gluten analysis, currently there is no certified reference material and moreover no suitable reference material is available for the detection of fermented-hydrolyzed gluten (9, 58, 97–100). Wheat gluten was chosen as a calibrant in order to avoid excluding any gluten protein fraction (gliadins or glutenins) from the analysis. Further, the material forms the regulatory basis for the analytical methods employed by the FDA (and several other governments) in assessing gluten content and potential health risk.

Using the multiplex-competitive ELISA, it was possible to distinguish between the wheat beers, barley beers, sourdough breads, and the soy-based sauces using cluster analysis. Of the 26 barley beers analyzed, 25 clustered separately from wheat beers and 24 clustered separately from sourdough breads. Only one barley beer clustered with the majority of soy-based sauces. It was also possible to distinguish samples with similar composition or processing within a particular category of fermented-hydrolyzed food by this method (e.g., some barley beers and gluten-reduced beers) (56). The various antibodies used in the multiplex-competitive ELISA may display different cross-reactivity patterns with wheat gluten, barley hordein, and rye secelin. However, these differences don't affect the utility of the multiplex-competitive ELISA. The classification of the peptide profiles is based on empirical observations and as such would appropriately group the fermented-hydrolyzed foods and accordingly enable the proper choice of reference materials that fit the empirical observation.

**Figure 4:**
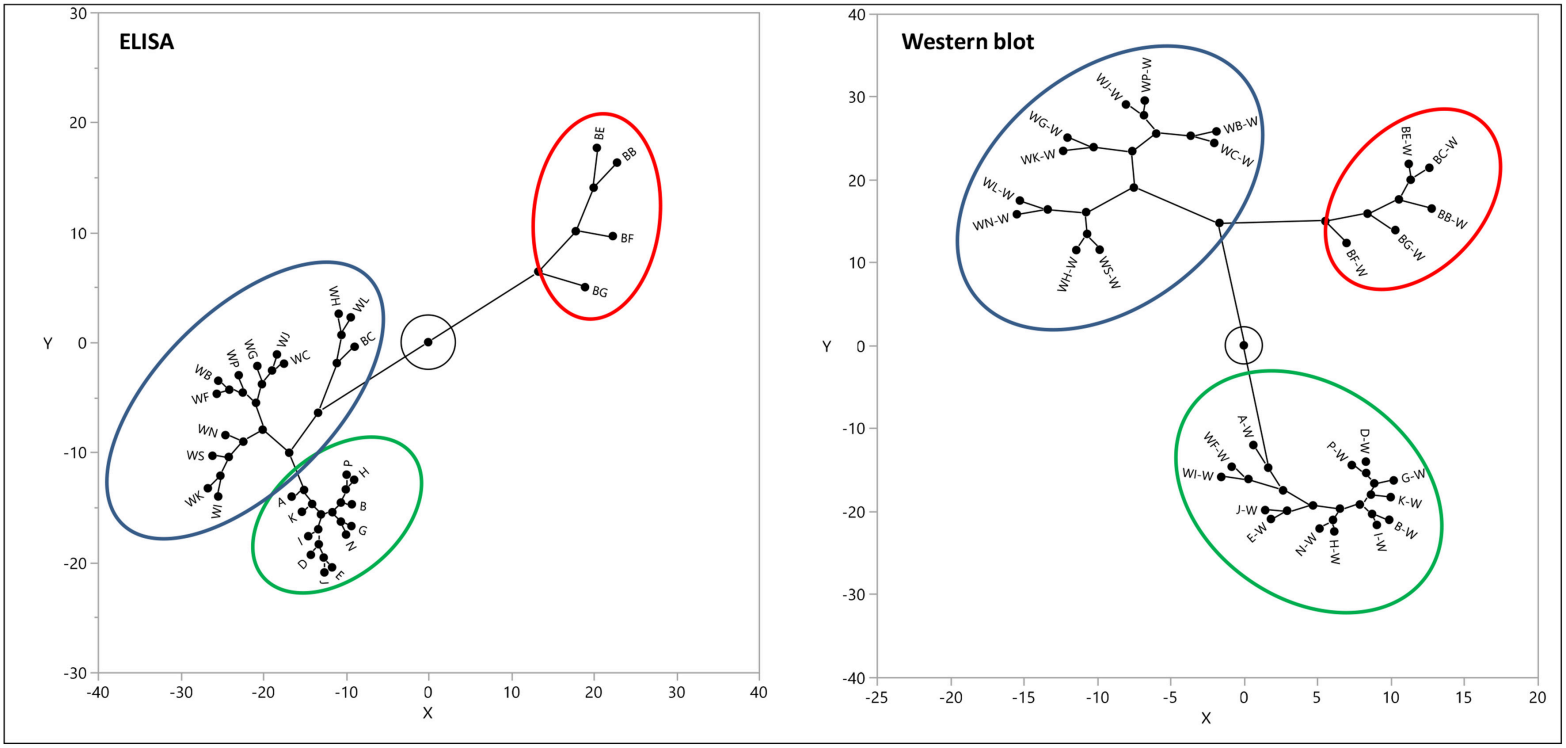
Constellation plot, displaying the clusters, of the apparent gluten concentration values (ppm) obtained by the multiplex-competitive ELISA and the estimated gluten concentration values obtained by western blot for 11 barley beers, 12 wheat beers, and 5 sourdough breads (49, 56). Samples analyzed by western blot have been identified by adding “W” after each sample code.

Western blot analysis utilizing the same gluten specific antibodies used in the multiplex-competitive ELISA confirmed the cluster analysis by the multiplex-competitive ELISA (49, 56). Although soy-based sauces showed non-specific false positive responses with the multiplex-competitive ELISA, it didn't affect the cluster pattern and the assay was still able to differentiate the soy-based sauces from other fermented-hydrolyzed foods. Indeed, the western blot analyses differentiated the false positive responses of soy-based sauces from the presence of antigenic proteinaceous materials. Figure 4 shows a constellation plot illustrating three different clusters that the barley beers, wheat beers and the sourdough bread generated when analyzed using the multiplex-competitive ELISA and western blot analyses, illustrating the ability of the assays to differentiate the protein/peptide profile characteristic of these three different fermented-hydrolyzed foods, which is essential for selection of appropriate calibration standard specific for each category of fermented-hydrolyzed foods required for accurate gluten quantitation.

It is obvious that further research is needed before accurate quantitation of gluten content in fermented-hydrolyzed foods can be achieved. The multiplex-competitive ELISA provides a first step by making it possible to determine the suitability of different hydrolysates as calibration standards for different fermentation-hydrolysis processes. This method also helps rule out false negative results. For examples, in Figure 2A, the apparent gluten concentration values of gluten-reduced beers with the two RIDASCREEN R5 antibodies and the two G12 antibodies were lower compared to the two Neogen Veratox antibodies and the Skerritt antibody. When the G12 or the RIDASCREEN R5 antibodies are used alone, the gluten content of the gluten-reduced beers may seem to be very low; however, the values are higher with both the Neogen Varatox antibodies and the Skerritt antibody, indicating that gluten components reactive to these antibodies are present at higher concentrations in these beers. Another potential utility of the multiplex-competitive ELISA especially with regulatory implications is in classification of an unknown fermented-hydrolyzed food sample into a particular category based on its overall apparent gluten concentration profile, subsequently allowing for the selection of an appropriate calibration standard required for accurate gluten analysis.

## Conclusion

It is currently impossible to accurately quantitate gluten in fermented/hydrolyzed foods and assess its potential immunopathogenicity using antibody-based methods. This is complicated by the fact that no current commercial antibody-based assay targets all the components of gluten. Further complicating the quantitative analysis of hydrolyzed gluten is the lack of appropriate calibrants that reflect the protein/peptide profiles characteristic of the various forms of fermentation. It is therefore necessary to first distinguish between the protein/peptide profiles to ensure the use of appropriate calibration standards for accurate quantitation. Mass spectrometry has potential by virtue of its ability to directly detect the peptides and proteins; however, its use as a routine analytical method is still in its infancy. In the meantime, the multiplex-competitive ELISA along with the western blot analysis make it possible to distinguish between the different protein/peptide profiles resulting from different fermentation processes and, ultimately, select appropriate standards for calibration.

## Author Contributions

EG oversaw the project. RP carried out the experiments, wrote the manuscript. All authors discussed the results and contributed to the final manuscript.

### Conflict of Interest Statement

The authors declare that the research was conducted in the absence of any commercial or financial relationships that could be construed as a potential conflict of interest.
